# Electrochemical Functionalization of Graphene‐on‐Cu(111): Reactivity, Onset Potentials, and Mechanistic Insights

**DOI:** 10.1002/advs.202501798

**Published:** 2025-04-15

**Authors:** Minhyeok Kim, Yongchul Kim, Geunsik Lee, Rodney S. Ruoff, Sun Hwa Lee

**Affiliations:** ^1^ Center for Multidimensional Carbon Materials (CMCM) Institute for Basic Science (IBS) Ulsan 44919 Republic of Korea; ^2^ Department of Chemistry Ulsan National Institute of Science and Technology (UNIST) Ulsan 44919 Republic of Korea; ^3^ Department of Materials Science and Engineering Ulsan National Institute of Science and Technology (UNIST) Ulsan 44919 Republic of Korea; ^4^ School of Energy and Chemical Engineering Ulsan National Institute of Science and Technology (UNIST) Ulsan 44919 Republic of Korea

**Keywords:** electrochemical functionalization, onset potential, reactivity of graphene, selective reaction, single‐crystal graphene

## Abstract

Electrochemical functionalization of graphene facilitates simple and various modifications of graphene properties. However, the scope of the available functional groups and the electrochemical behavior of graphene is not fully understood. The electrochemical reactivity of single crystal and monolayer graphene‐on‐Cu(111) with various phenyl and alkyl iodides is investigated, and discovered different onset potentials, identifying the extent of the reaction with Raman spectroscopy and X‐ray photoelectron spectroscopy (XPS). Differential pulse voltammetry (DPV) and density functional theory (DFT) calculations are employed to elucidate the onset potential differences between the phenyl iodides with different substituents. A comprehensive understanding of graphene's electrochemical reactivity is presented.

## Graphical abstract

The electrochemical reactivity of graphene‐on‐Cu(111) with various phenyl and alkyl iodides showed different onset potentials. Differential pulse voltammetry (DPV) and density functional theory (DFT) calculations are used to elucidate the differences in onset potentials for phenyl iodides with different substituents. A comprehensive understanding of graphene's electrochemical reactivity is presented.

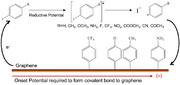

## 1 Introduction

Graphene has been functionalized using diazonium salts,^[^ ^1^, ^2^, ^3^, ^4^, ^5^, ^6^, ^7^, ^8^, ^9^ ^]^ alkali metal alloys,^[^ ^10^, ^11^, ^12^, ^13^ ^]^ and photochemical^[^ ^14^, ^15^, ^16^, ^17^, ^18^, ^19^ ^]^ or electrochemical reactions.^[^ ^20^, ^21^, ^22^, ^23^ ^]^ While there are some reports^[^ ^11^, ^24^, ^25^ ^]^ that the presence of mechanical stress between graphene and substrate (thus, non‐zero strain in graphene) can enhance the reactivity of graphene, the chemical functionalization of graphene has typically been done with a highly reactive reagent,^[^ ^26^ ^]^ e.g., radical species or by adding charge to graphene (such as by using NaK), to overcome the inherent stability of the carbon lattice and functionalize the graphene.

The covalent functionalization of graphene yields graphene derivatives with stable structures,^[^ ^26^ ^]^ whose properties, e.g., band gap,^[^ ^4^, ^7^, ^27^, ^28^, ^29^ ^]^ electrical doping,^[^ ^30^, ^31^, ^32^ ^]^ wettability,^[^ ^33^, ^34^ ^]^ and catalytic effects,^[^ ^35^, ^36^, ^37^ ^]^ can be manipulated.

We reported a simple and efficient approach to electrochemically functionalize graphene using 4‐iodoaniline.^[^ ^23^ ^]^ External influences on the reactivity of graphene, e.g., lattice defects, grain boundaries, and contamination arising due to transfer from a growth substrate, were avoided by using as‐grown single‐crystal monolayer graphene on Cu(111) foils as the working electrode. We found^[^ ^23^ ^]^ a relatively small surface potential gap for graphene atop Cu(111) compared to atop Cu(115); Cu(115) can be present in “single crystal” Cu(111) foils as small twin regions. Having thereby learned that even a small potential change significantly altered the reactivity of graphene to 4‐iodoaniline, we were inspired to undertake the study reported here with graphene‐on‐Cu(111) and a wide range of molecules.

We have not found published studies where a broad range of molecules as reagents have been studied. Also, almost invariably, graphene has first been transferred to another substrate, which can potentially introduce impurities.^[^ ^38^ ^]^ Here, we study a broad range of phenyl and alkyl iodides and avoid contamination‐from‐transfer by using single‐crystal graphene as directly CVD grown on Cu(111) foils. Aryl diazonium compounds undergo spontaneous covalent functionalization of graphene, so we selected aryl and alkyl halides that do not spontaneously react: the electrochemical driving force could then be studied.

Raman spectroscopy and X‐ray photoelectron spectroscopy (XPS) were used to investigate the degree of functionalization (at room temperature in a particular electrochemical solvent/electrolyte system described below). We found the reactivity differs by measuring the onset potential for reaction as a function of the reagent type. Differential pulse voltammetry (DPV) and density functional theory (DFT) calculations were done to try to understand the measured onset potentials. To further study the reactivity of graphene‐on‐Cu(111), the selectivity of the reaction was explored using two molecules that showed a large difference in the onset potential.

We anticipate that this study will serve as a foundation for further studies of the electrochemical behavior of graphene and exploration of a broader range of graphene properties.

## 2 Results and Discussion

**Figure 1:**
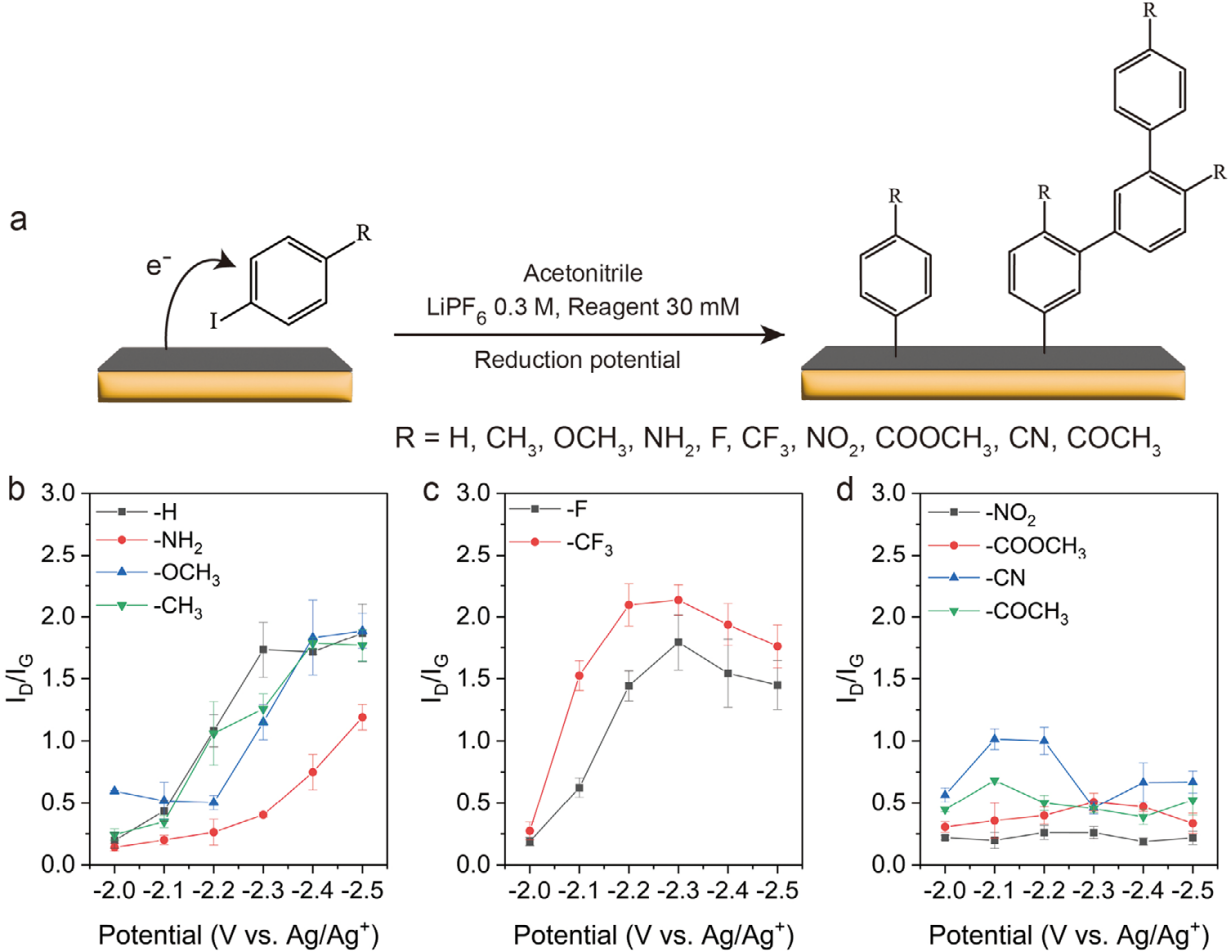
a) A scheme of electrochemical functionalization. The I_D_/I_G_ plot of functionalized graphene reacted with b) iodobenzene, 4‐iodoaniline, 4‐iodoanisole, 4‐iodotoluene, c) 4‐fluoroiodobenzene, 4‐iodobenzotrifluoride, and d) 1‐iodo‐4‐nitrobenzene, methyl 4‐iodobenzoate, 4‐iodobenzonitrile, and 4′‐iodoacetophenone.

We used a 3‐electrode system with single‐crystal monolayer graphene on Cu(111) as a working electrode, Pt as a counter electrode, and an Ag/Ag^+^ reference electrode. The cyclic voltammetry (CV) technique was used to compare the reactivity of graphene toward 10 different phenyl iodide molecules. The potential range was from ‐1.0 V to the maximum potential displayed on the x‐axis (from ‐2.0 to ‐2.5 V), and the scan rate was 100 mV s^−1^. After 20 cycles, the intensity ratio of the D band (≈1360 cm^−1^) and G band (≈1590 cm^−1^) (I_D_/I_G_) was obtained from Raman spectra measured at random points, indicating the degree of functionalization.^[^ ^38^ ^]^ We note that polyaryl formation could occur when functionalizing graphene with aryl compounds,^[^ ^28^, ^39^ ^]^ as shown in **Figure** **1**a.

The I_D_/I_G_ ratio increased by increasing potential for iodobenzene and phenyl iodide that were substituted with electron‐donating groups (EDGs), 4‐iodotoluene, 4‐iodoanisole, and 4‐iodoaniline(─CH_3_, ─OCH_3_, and ─NH_2_, Figure 1b), or electron‐withdrawing groups (EWGs): 4‐fluoroiodobenzene and 4‐iodobenzotrifluoride (4‐IBTF) (─F, ─CF_3_, Figure 1c). The EWG‐functionalized graphene had relatively higher I_D_/I_G_ values than the EDG‐functionalized graphene at the same applied potential. In contrast, the I_D_/I_G_ ratio did not increase for EWGs that have double or triple bonds: 1‐iodo‐4‐nitrobenzene, methyl 4‐iodobenzoate, 4‐iodobenzonitrile, and 4′‐iodoacetophenone (─NO_2_, ─COOCH_3_, ─CN, and ─COCH_3_, Figure 1d). 4‐iodobenzonitrile and 4′‐iodoacetophenone showed a slight increase of I_D_/I_G_ at a relatively low potential, but I_D_/I_G_ did not increase at a larger potential.

Thus, two factors affect the electrochemical reactivity of graphene: i) the substituent and ii) the existence of double or triple bonds on the substituent. As mentioned, among these reagents, the EWGs have a higher reaction rate than EDGs. We suggest this is because EWGs generate an electron‐deficient phenyl radical intermediate, increasing reactivity with the negatively charged graphene.^[^ ^40^ ^]^ The EWGs with double or triple bonds were not functionalized; we suggest such molecules form “resonance structures” as the reduced species (thereby stabilizing them). This could lead to side reactions instead of the C‐I cleavage required to covalently bond with graphene, for example, coupling with other reagents or solvent molecules.

We observed “exceptional behavior” from 4‐iodobenzonitrile and 4′‐iodoacetophenone(Figure 1d). These had small increases of I_D_/I_G_ at certain potentials. One possible explanation is that side reactions occur with the molecules having a strong triple bond (─CN) or a weak resonance (─COCH_3_) at a larger potential, but they could covalently bond with graphene at an appropriate potential at room temperature.

Alkyl iodides and those with heteroatoms (O or N) were explored to compare their reactivities (Figure S2, Supporting Information). The I_D_/I_G_ increased with short alkyl iodides, iodomethane, and iodoethane, while it did not with the longer alkyl iodides and those having O and N. This could be because the long alkyl iodides, when they are reduced, can undergo an intra‐ or intermolecular reaction such as hydrogen atom transfer, radical rearrangement, or dimerization.^[^ ^41^, ^42^, ^43^, ^44^ ^]^

**Figure 2:**
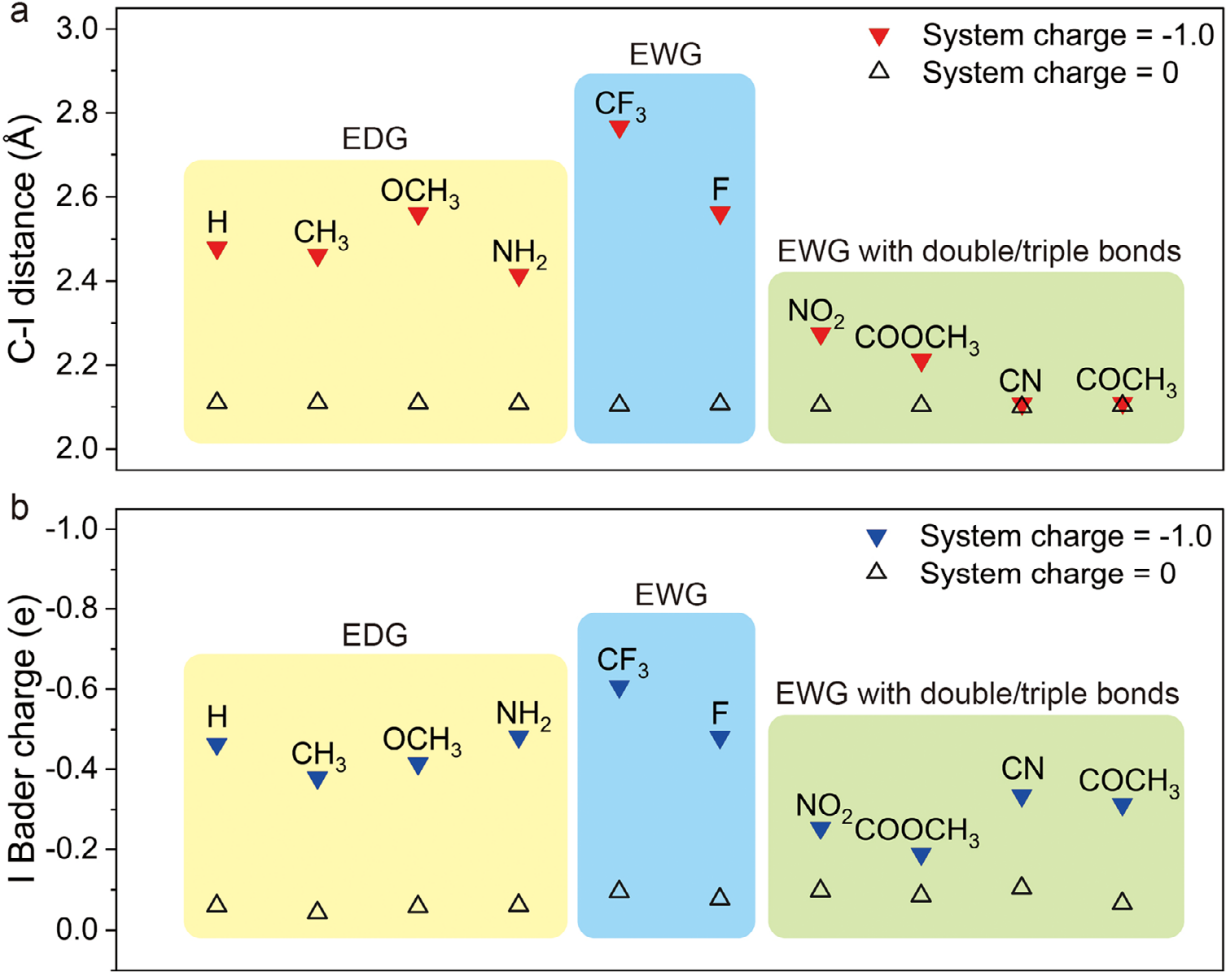
a) DFT calculation results for the C‐I distance and b) the Bader charge of iodine for the aromatic molecules as the system charge decreases from 0 to ‐1.0 e. Detailed C‐I distance and Bader charge results are shown in Figure S3 (Supporting Information), and the molecular structures are shown in Figure S4 (Supporting Information).

Through DFT calculations, we varied the system charge and optimized the structures of reagent molecules involved in functionalization to calculate the C─I bond length (**Figure** **2**a) and Bader charge (Figure 2b) in our attempt to understand the reduction process.

At a system charge of ‐1.0 e, the C‐I distance and the Bader charge increased for the molecules that showed I_D_/I_G_ increasing. The 4‐IBTF particularly showed a larger change in the C─I bond distance and Bader charge. Conversely, smaller C‐I distances and lower Bader charges were observed in the EWGs with double or triple bonds that did not form covalent bonds with graphene. The C‐I distance changes were negligible with the molecules having “exceptional behaviors,” but the Bader charge changes were comparable to some of the EDG molecules.

For the EDGs and EWGs, Bader charge analysis shows that iodine readily attracts electrons and the optimized structure exhibits a notably longer C─I bond length, indicating that the C─I bond may readily dissociate when the reduction potential is applied.

In contrast, increases in system charge do not significantly extend the C─I bond length in the EWG with double/triple bonds. A weak dipole moment is formed despite additional electron doping, suggesting that bond weakening under applied potential is relatively insignificant. Relatively small Bader charge increases imply that the electrons can be delocalized when the molecule is reduced, making the C‐I cleavage difficult.

The 4‐iodobenzonitrile and 4′‐iodoacetophenone formed a small degree of covalent bonding at certain potentials, while they have stronger C─I bonds to the molecules in B. We suggest one should consider the possibility of alternative reaction pathways of covalent functionalization through heteroatoms (e.g., C─N or C─O) instead of the formation of a C─C bond between these reagents and graphene.

Large reactivity differences were observed among the reagents. 4‐iodoaniline showed relatively lower I_D_/I_G_ change, while 4‐fluoroiodobenzene and 4‐IBTF showed larger I_D_/I_G_, compared to iodobenzene and 4‐iodotoluene which have a similar increasing rate (Figure 1b,c). We found that this difference can be estimated by an “onset potential”, the applied potential at which a measurable degree of functionalization occurs.

**Figure 3:**
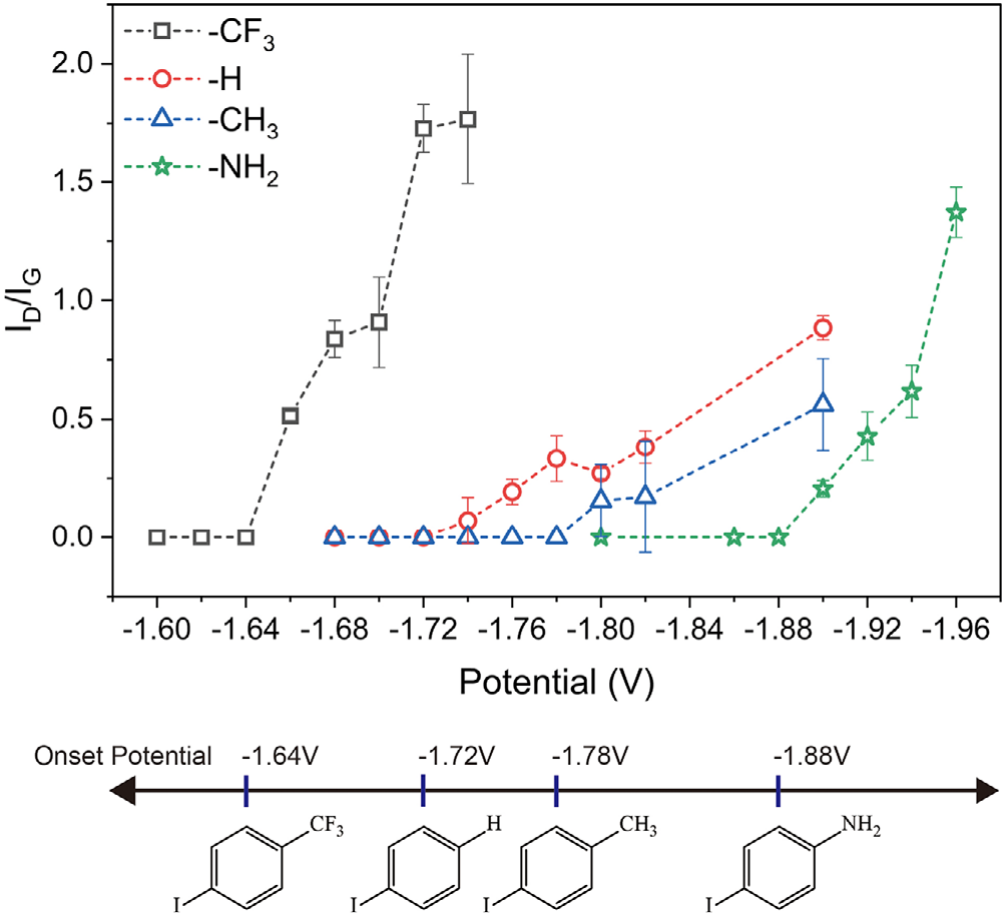
I_D_/I_G_ ratio change of 4‐IBTF, iodobenzene, 4‐iodotoluene, and 4‐iodoaniline after electrochemical functionalization at each potential for 30 min.

We chose 4‐IBTF, iodobenzene, 4‐iodotoluene, and 4‐iodoaniline (**Figure** **3**) to compare the onset potential difference since they have a clear difference in reactivity, as seen in Figure 1. A constant potential was applied for 30 min at every 0.02 V. These applied potentials were correlated with measured Raman spectra.

The D bands gradually decreased as the potential decreased and then disappeared when the potential reached a certain level, which we call the onset potential.

Measuring an accurate I_D_/I_G_ ratio at a low degree of functionalization is challanged by the background noise from fluorescence from the Cu substrate. We filtered the I_D_/I_G_ value when the D band intensity was too small so that the signal‐to‐noise value was less than a threshold value. We explain this filtering in Figure S1 (Supporting Information).

The obtained onset potentials were ‐1.64 V(4‐IBTF), ‐1.72 V(iodobenzene), ‐1.78 V(4‐iodotoluene), and ‐1.88 V(4‐iodoaniline). The reagent with a larger reactivity has a lower onset potential. As mentioned, different reactivities of single‐crystal graphene on Cu(111) and Cu(115) regions were observed with a difference of 0.06 V.^[^ ^37^ ^]^ The onset potential difference of 0.24 V between 4‐IBTF and 4‐iodoaniline is comparatively large and can be finely controlled to modify the reaction rate.

**Figure 4:**
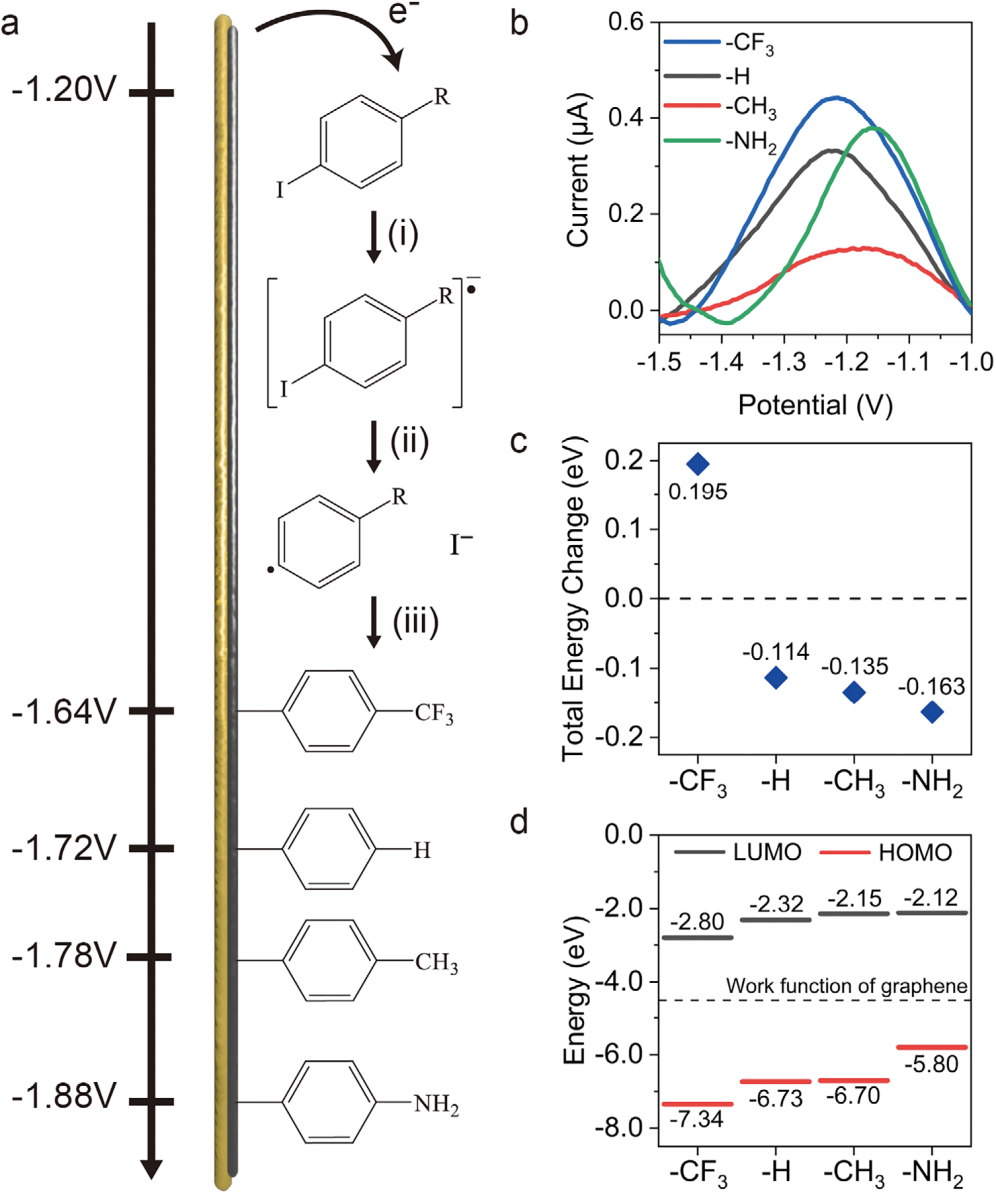
a) A proposed reaction pathway scheme. b) DPV curve obtained from iodobenzene, 4‐iodotoluene, 4‐IBTF, and 4‐iodoaniline. DFT calculation results for c) the total energy change of the dissociation process from molecular anion to phenyl radical and iodide ion and d) the highest occupied molecular orbital (HOMO) and lowest unoccupied molecular orbital(LUMO) energy diagram of phenyl radicals. The dashed line is the work function of graphene.

Considering the reduction of the aryl halides^[^ ^45^, ^46^ ^]^ (see **Figure** **4**a), we propose: i) a heterogeneous electron transfer (HET) occurs as a reagent is reduced, followed by ii) dissociation into an aryl radical and an iodide anion, and then iii) the aryl radical covalently bonds to graphene, which is an irreversible process.

Redox current with reduced background signals can be obtained using the DPV technique to investigate step i) (see Figure 4b). We observed the reduction peaks at near ‐1.20 V from all the reagents.

DFT was employed to calculate the energy change (see Figure 4c) from the reduced molecular anion to the aryl radical with the iodide ion for step ii), and the ionization energy(IE) of the radicals (see Figure 4d) for step iii). The calculated energy changes were 0.195 eV (4‐IBTF), ‐0.114 eV(iodobenzene), ‐0.135 eV (4‐iodotoluene), and ‐0.163 eV (4‐iodoaniline). The IE can be estimated from the HOMO energy level, which is related to the feasibility of forming a covalent bond with graphene, corresponding to step iii). The benzotrifluoride radical has the highest IE, whereas the aniline radical has the lowest, and benzene and toluene radicals have similar IE values.

The experimental C‐I reduction peak positions were significantly lower than the onset potentials. Considering the noticeable color change of the 4‐iodoaniline solution at ‐1.70 V without observing a D band increase (Figure S5, Supporting Information), this implies that the HET step occurred, but graphene was not functionalized. In other words, the HET step does not determine the onset potential or the reactivity of graphene.

Thus, we suggest the functionalization of graphene includes at least two separate steps in which an intermediate is produced. The intermediate could make side reactions, which might be preferred to the functionalization of graphene when insufficient energy to form the C─C covalent bond to graphene is available.

We can roughly compare the stability of the radicals from the energy change from the reduced molecule to the aryl radical and iodide ion (Step ii in Figures 4a, and 4c). The radical produced from 4‐IBTF should have lower stability than others, while 4‐iodoaniline produces a more stable radical. Since a stable radical species could have a long diffusion length, increased stability of radicals may hinder the formation of covalent bonds by providing time for radicals to diffuse away from the surface. From this perspective, an unstable radical is expected to be more reactive to graphene.

When a radical is formed, the HOMO level is in a half‐filled state, which enables it to participate in graphene functionalization by accepting electrons as it bonds to the surface. This electron transfer fills the HOMO level, facilitating functionalization. A larger energy difference between the valence band maximum (VBM) of graphene and the radical's HOMO level thus yields easier bond formation. A covalent bond formation can be facilitated if a phenyl radical has a larger IE compared to graphene because graphene can more readily provide electrons to make bonds.^[^ ^40^ ^]^ The total energy change and the IE explain the onset potential difference.

While experimental and theoretical results support this suggested reaction mechanism, additional studies are indicated. The DFT calculations do not include the actual electrochemical system, for example, the interface, electric field, solvent, or charge transfer processes.

The selectivity of the reaction was assessed by the difference in onset potential. Functionalization by a reagent with a lower onset potential is expected to be preferred to that with a higher onset potential if a reaction solution containing various reagents is used.

We used a reaction solution with 4‐IBTF(3 mm) and 4‐iodoaniline(30 mm), whose onset potentials are ‐1.64 V and ‐1.88 V, respectively, and applied ‐1.56 V, ‐1.80 V, and ‐1.96 V for 60 min. We used a molar ratio of 1:10 to control the reaction rate of the 4‐IBTF because the molar ratio of 1:1 produced a functionalized graphene significantly dominated by 4‐IBTF (Figure S6, Supporting Information).

**Figure 5:**
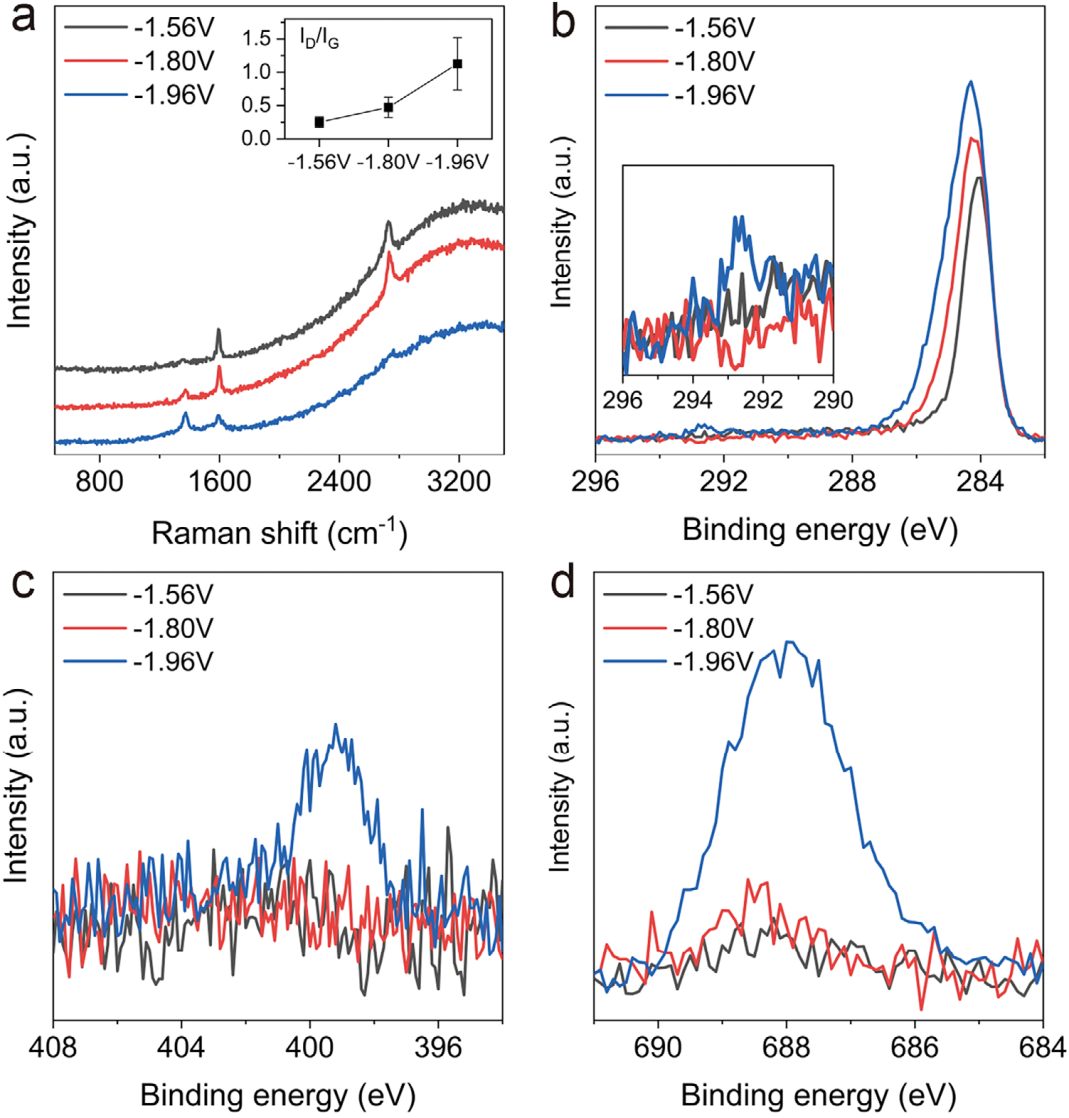
a) Raman spectra of the functionalized graphene reacted at ‐1.56, −1.80, and ‐1.96 V for an hour with the reaction solution containing both 4‐iodoaniline and 4‐IBTF. High‐resolution XPS spectra of b) C1s, c) N1s, and d) F1s of each sample were obtained after etching with Ar cluster ion sputtering for 320 s.

Raman spectra showed negligible D band intensity at ‐1.56 V. The D band intensity increased for ‐1.80 V and ‐1.96 V (**Figure** **5**a).

XPS spectra were acquired to elucidate the extent of reaction. 2 keV Ar cluster ions were sputtered on the surface for 320 s before each measurement to remove surface adsorbates. We observed a broadening of the C1s spectrum due to, e.g., C─C sp^3^ bonds (284.8 eV), C─N and C─O bonds (286.0 eV), and C─CF (285.4 eV) bonds. The C─F (292.4 eV) signal appeared when ‐1.96 V was applied (Figure 5b).

The N1s signal was not detected at ‐1.56 V and ‐1.80 V, while a small peak was observed at 399.4 eV when ‐1.96 V was applied (Figure 5c). Similarly, no F1s signal was detected at ‐1.56 V and ‐1.80 V, but a large peak at 688.0 eV was observed when ‐1.96 V was applied (Figure 5d). Although the potential was larger than the onset potential of 4‐IBTF, the F1s signal at ‐1.80 V was not detected, As we extended the reaction time (2 h and 3 h) at ‐1.80 V, the F1s signal did not significantly change (Figure S7, Supporting Information). This is attributed to the low degree of functionalization due to the low concentration of 4‐IBTF.

The degree of functionalization increased with the applied potential. Even with the 1:10 molar ratio, the F1s signal was much larger than the N1s, implying that 4‐IBTF dominates when ‐1.96 V was applied. 4‐IBTF dominated the reaction when 4‐iodoaniline was contained in the same reaction solution. It should be noted that decreasing the molar ratio of 4‐IBTF to 4‐iodoaniline from 1:1 to 1:10 did not increase the degree of functionalization by 4‐iodoaniline (Figure S6, Supporting Information). This demonstrates that single‐crystal graphene‐on‐Cu(111) can be selectively functionalized at room temperature by a reagent with a lower onset potential.

## 3 Conclusion

We measured the electrochemical reactivity of single crystal monolayer graphene‐on‐Cu(111) toward various phenyl iodides and alkyl iodides. Raman spectroscopy showed that the reagents with substituents containing double or triple‐bonds or long‐chain‐shaped alkyl iodides did not functionalize graphene. Reagents that did functionalize graphene had different onset potentials, and those with smaller onset potentials showed larger reactivity at the same applied potential.

We proposed a reaction pathway that can explain the difference in reactivities, based on DPV analysis and DFT calculations; DPV analysis showed that reduction of the C─I bond does not determine the onset potential, and the DFT calculation showed the total energy change and ionization energy of the radicals rationalizes why the reactivities were ordered according to the onset potential. Finally, we examined the selectivity of the electrochemical reaction of graphene‐on‐Cu(111) when two different reagents were present at the same time, finding that the graphene was predominantly functionalized by the reagent with a smaller onset potential, even when it was at ten times lower concentration than the other reagent.

The electrochemical method can typically be done with mild reaction conditions and is scalable for applications. By demonstrating that each reagent has a distinct “onset potential” for covalent bonding to graphene, our work highlights a key aspect of the reaction mechanism, elucidating how graphene interacts with likely intermediate species under electrochemical conditions.

## Conflict of Interest

The authors declare no conflict of interest.

## Supporting information



Supporting Information

## Data Availability

The data that support the findings of this study are available from the corresponding author upon reasonable request.
