# Supplementary material for: Electrochemical Functionalization of Graphene‐on‐Cu(111): Reactivity, Onset Potentials, and Mechanistic Insights
Source: Adv Sci (Weinh). 2025 Apr 15;12(26):2501798. doi: 10.1002/advs.202501798 (PMC12245042; doi:10.1002/advs.202501798)
Supplement: Supplementary file 1 — Supporting Information [file ADVS-12-2501798-s001.docx]

**Supplemental Information**

**Electrochemical Functionalization of Graphene-on-Cu(111): Reactivity, Onset Potentials, and Mechanistic Insights**

*Minhyeok Kim^1,2^, Yong-Chul Kim^1^, Geun-Sik Lee^2^, Rodney S. Ruoff^1,2,3,4^*, Sun Hwa Lee^1^**

^1^Center for Multidimensional Carbon Materials (CMCM), Institute for Basic Science (IBS), Ulsan 44919, Republic of Korea

^2^Department of Chemistry, School of Natural Science, Ulsan National Institute of Science and Technology (UNIST), Ulsan 44919, Republic of Korea

^3^Department of Materials Science and Engineering, Ulsan National Institute of Science and Technology (UNIST), Ulsan 44919, Republic of Korea.

^4^School of Energy and Chemical Engineering, Ulsan National Institute of Science and Technology (UNIST), Ulsan 44919, Republic of Korea.

Corresponding authors:

Rodney S. Ruoff, Email: ruofflab@gmail.com or rsruoff@ibs.re.kr

Sun Hwa Lee, Email: sunhwa.lee82@gmail.com or sunhlee@ibs.re.kr

Keywords: Electrochemical functionalization, Single-crystal graphene, Onset potential, Reactivity of graphene

## Experimental Methods

**Preparation of graphene**

Cu(111) foil was prepared by annealing Cu foil (99.99%, 50 μm thickness, Nilaco Co., Japan) in a quartz tube furnace. After annealing at 1050 °C for 18 h with flowing Ar and H_2_(100 sccm, 99.999%), the monolayer graphene was grown on it by chemical vapor deposition method. The temperature was held at 1070°C with a gas flow of Ar(99.999%, 300 sccm), H_2_(99.999%, 50 sccm), and CH_4_ (0.1% diluted in Ar, 35 sccm).

**Electrochemical functionalization**

The graphene (1 cm^2^, working electrode) and Pt plate (counter electrode) clipped by the Pt/PTFE electrode holder and Ag/AgNO_3_(0.01 M) reference electrode(acetonitrile, LiPF_6_ 0.1M) were assembled with a quartz cell in an Ar glovebox. The reaction solution was prepared with acetonitrile that had been purged with Ar for at least 2 hours. The supporting electrolyte, LiPF_6_ (>99.99%, 0.3 0M, Sigma-Aldrich), and the reagents (30 mM, Sigma-Aldrich) were stirred in a vial for at least 3 hours in the acetonitrile. The quartz cell was wrapped by aluminum foil to prevent unintended reactions because many of the iodo compounds are light-sensitive. CV and chronoamperometry methods were used to functionalize graphene. For the CV, the cycle number was 20 and the scan rate was 100 mV/s with the potential range from -1.0V to the noted values. After the reaction, the graphene samples were rinsed with acetone, isopropyl alcohol, and deionized water, and dried in a vacuum desiccator.

**Differential pulse voltammetry**

An electrode holder that seals the electrode area with only a small, exposed area was used. The graphene surface area was fixed at 0.07 cm^2^. The solution was prepared with the purged acetonitrile containing LiPF_6_ (0.10 M) and each reagent (4-IBTF, iodobenzene, 4-iodotoluene, or 4-iodoaniline, 100 μM). After each measurement, the quartz cell was cleaned by merging it in nitric acid (50 wt%, Sigma-Aldrich) for 30 min and piranha solution(H_2_O_2_:H_2_SO_4_ = 1:3, Daejeong and Sigma-Aldrich) for 30 min, and washing with DI water and ethanol. The measurement was performed with the pulse size 60 mV, step size 5 mV, and sample period 1 s with pulse time 0.05s, which had been optimized to obtain signals.

**Characterization**

Raman spectra were obtained with a WiTec alpha 300 M with a 488 nm laser at room temperature. XPS spectra were obtained with a Thermo Scientific ESCALAB-250Xi instrument. All electrochemical measurements and functionalization were done with a Gamry 600+.

**Filtering D bands in Raman spectra by a threshold signal-to-noise value**

I_D_/I_G_ value was filtered by a threshold signal-to-noise ratio (SNR).

$$SNR=\frac{S_{signal}-S_{noise}}{N_{rms}}$$

Where S_signal_ is the maximum value in the D band range, S_noise_ is the background noise measured in the 1200 – 1300 cm^-1^ range (where there are no Raman peaks; by using “asymmetric least squares smoothing” by Origin-Pro), and the *N_rms_* is the standard deviation of the background:

$$N_{rms}=\sqrt{\frac{\sum_{i}^{n} {(S_{i}-\bar{S})}^{2}}{n}}$$

The threshold value was determined by measuring the I_D_/I_G_ ratio of Raman spectra measured on single crystal graphene on Cu(111).


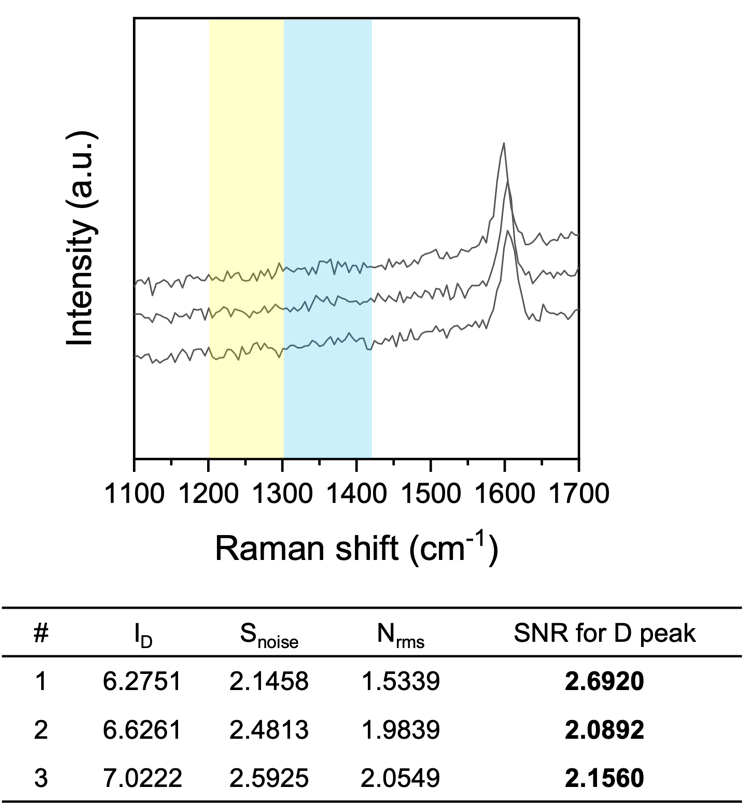


Figure S1. (top) Raman spectra measured on a pristine graphene-on-Cu(111) at 3 randomly chosen regions. The background and D peak regions were marked with yellow and blue boxes. (bottom) table of the maximum intensity of the D peak range, value of the obtained noise signal, N_rms_, and SNR value for the D peak range.

The average I_D_/I_G_ values were obtained from the Raman spectra shown on the left side in Figure S1. The SNR for D peaks of pristine graphene is between 2 and 3. Assuming the D band does not appear on the pristine graphene, we found that the SNR ratio should be larger than 3 to be able to filter the I_D_/I_G_ ratio correctly from the background noise. With SNR ≥ 3, the D band intensity would be filtered to 0, therefore, the I_D_/I_G_ would be 0. A proper threshold SNR could be different for a different spectrum. For example, the proper threshold SNR could be 2 or 6 depending on the measurement parameters that affect the noise level(*i.e*., laser power, accumulation time, integration number, etc.). In our experiments, we concluded that the proper threshold SNR is 5.

**Calculation Methods**

The DFT calculations were carried out by using Vienna ab initio simulation package (VASP)^[1]^ with the GGA-PBE type exchange-correlation functional.^[2]^ In order to describe the planewave energy cutoff of 600 eV. To capture the subtle variations arising from changes in system charge, a plane wave energy cutoff of 600 eV was employed with spin-polarization. Additionally, a large cubic cell with a lattice length of 30 Å was used to control the effects of compensating charges, and dipole corrections were applied in all directions. Structure optimization was carried out until the forces on all atoms were less than 0.01 eV/Å, with D3-Grimme^[3]^ included for dispersion correction. Only the Gamma point was used for k-point sampling, and the HSE06 hybrid functional^[4]^ was employed to accurately determine the HOMO and LUMO levels.


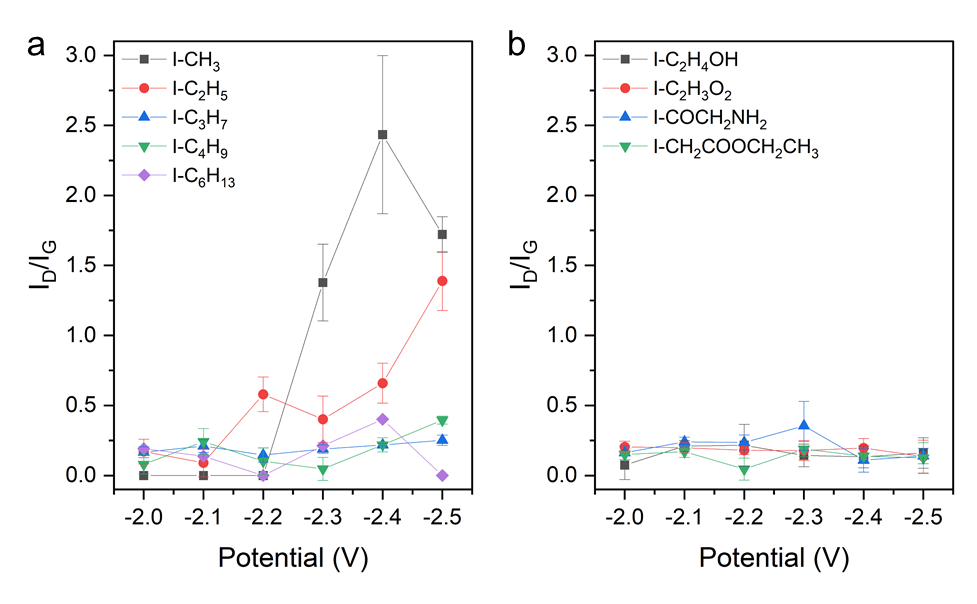


*Figure S2. The I_D_/I_G_ plot of functionalized graphene reacted with (a) iodomethane, iodoethane, 1-iodopropane, 1-iodobutane, 1-iodohexane and (b) iodoethanol, iodoacetic acid, iodoacetamide, ethyl iodoacetate.*


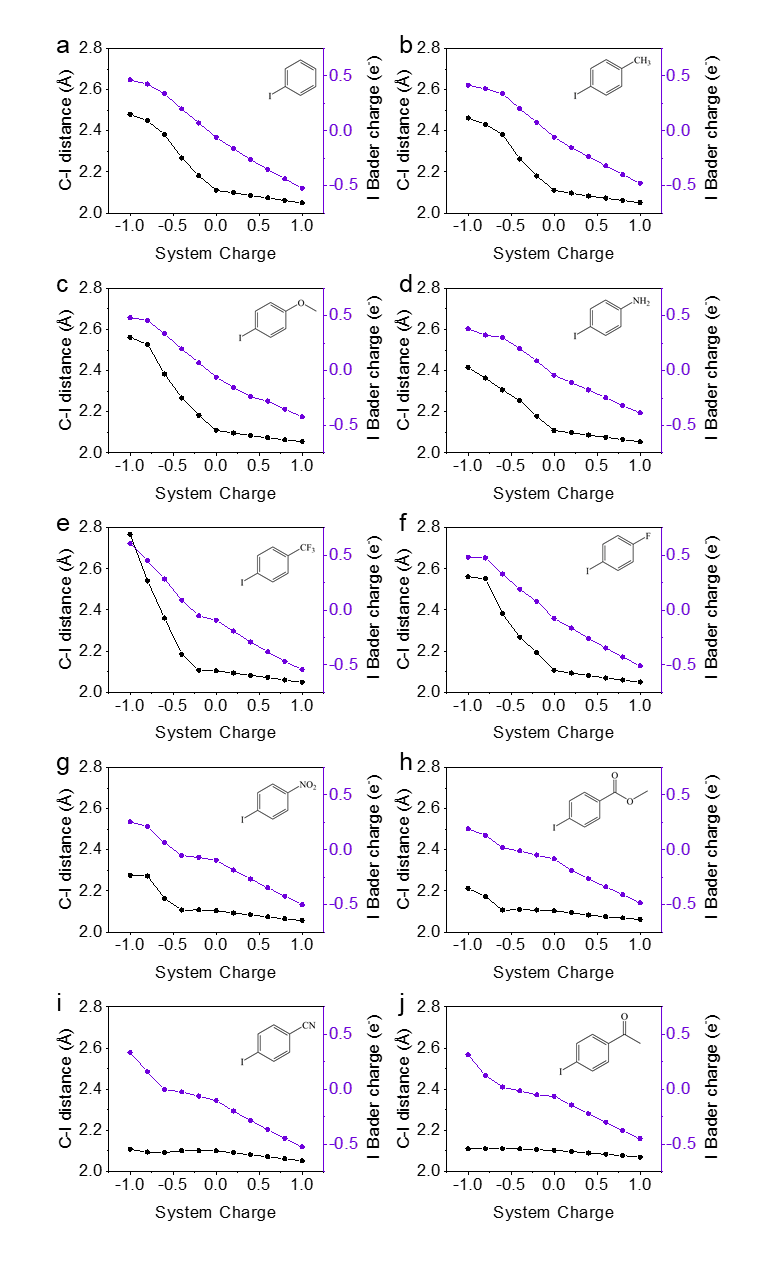


*Figure S3. DFT calculation results for the C-I distance and the Bader charge of the iodine atom by the system charge changing from -1.0 to 1.0 e.*

*
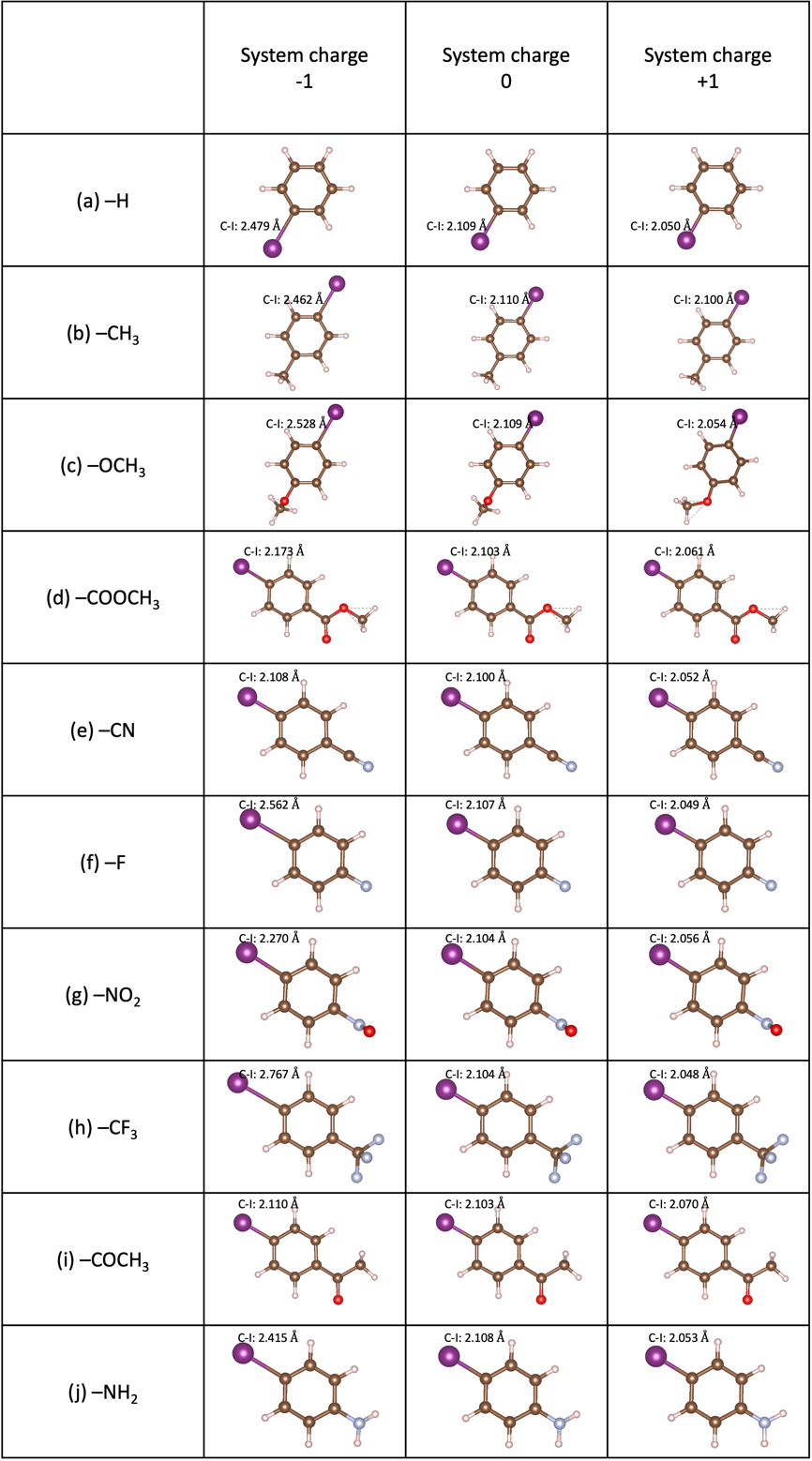
*

*Figure S4.* *The structure of the molecules with charges -1, 0, and +1*


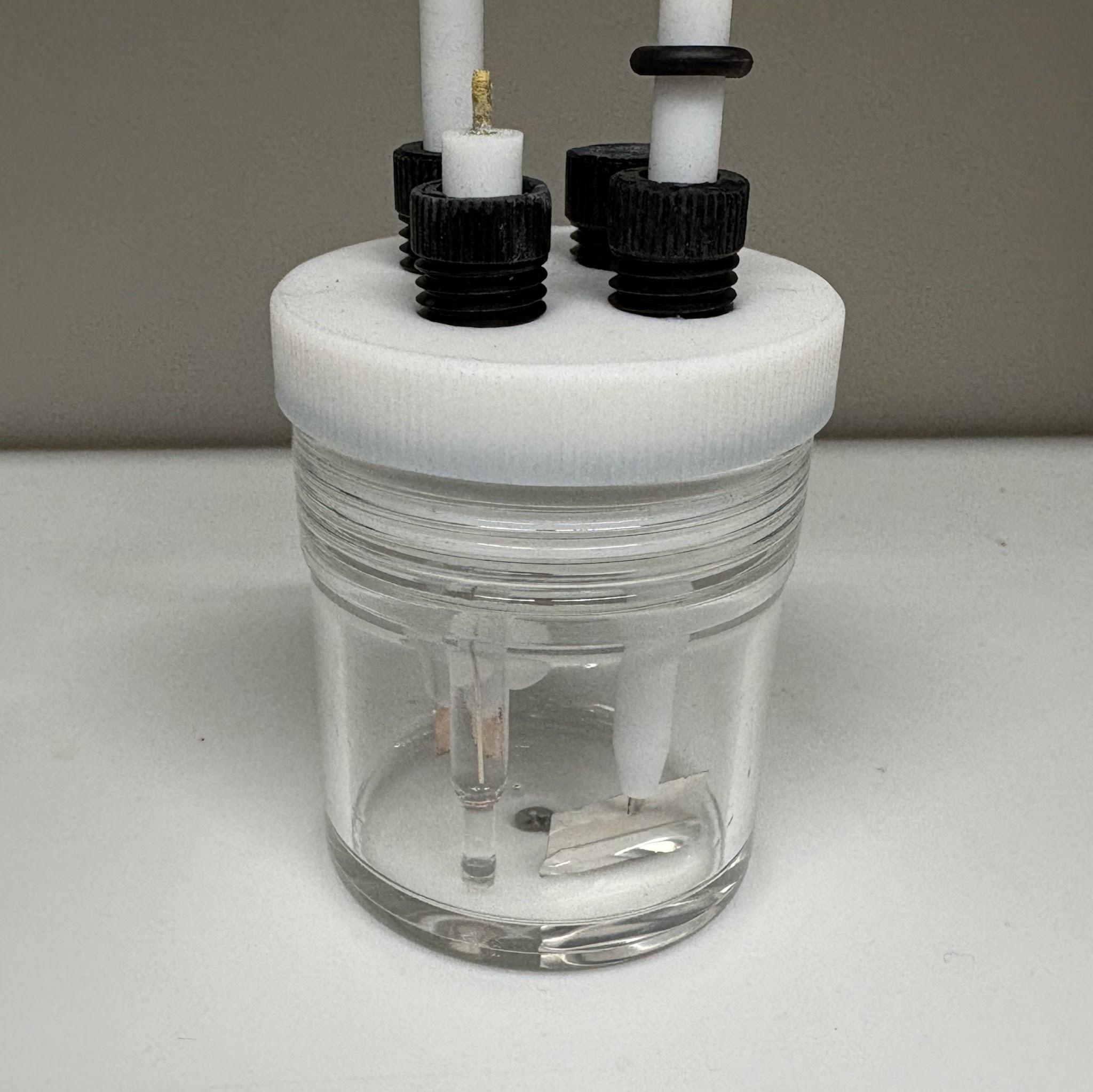

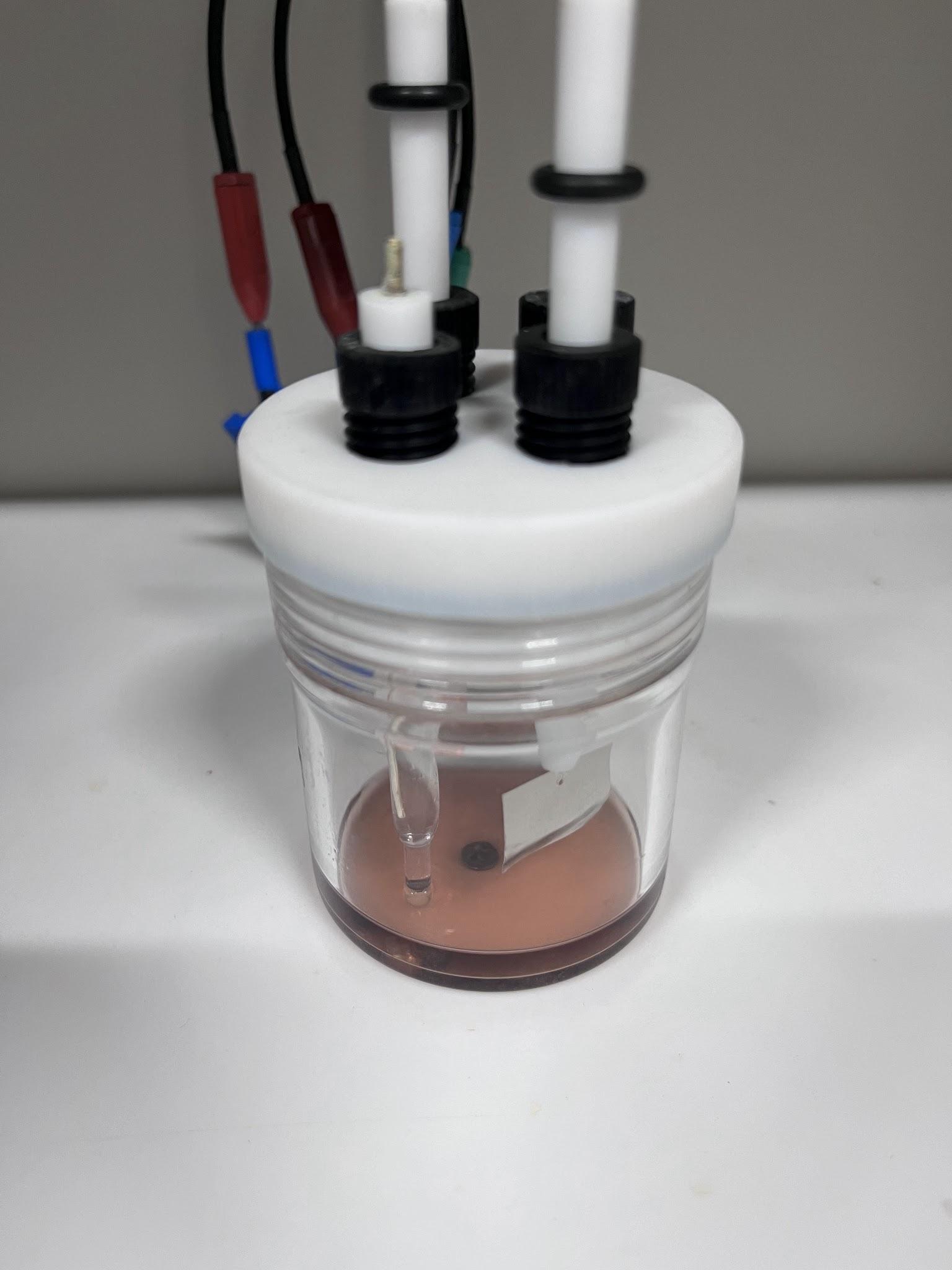


*Figure S5. A photograph of the reaction solution containing 4-iodoaniline and LiPF_6_ before (left), and after the electrochemical reaction at a constant potential of -1.70V for 60 min(right). The transparent solution changed to pink-brown after the reaction.*


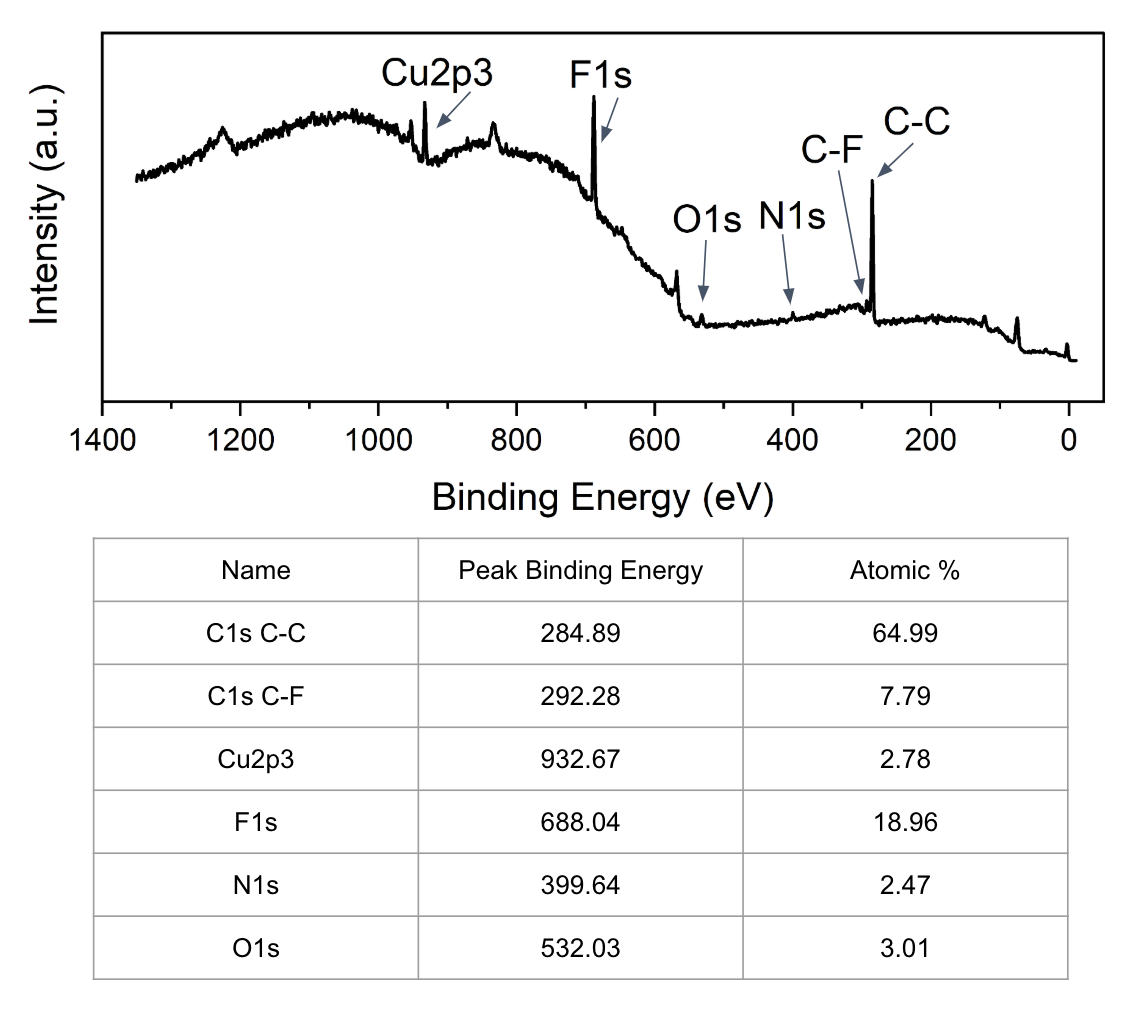


*Figure S6. XPS spectrum and the atomic ratio of the graphene functionalized by 4-iodoaniline and 4-IBTF with a concentration ratio of 1:1 at -1.96V for 60 min.*

*
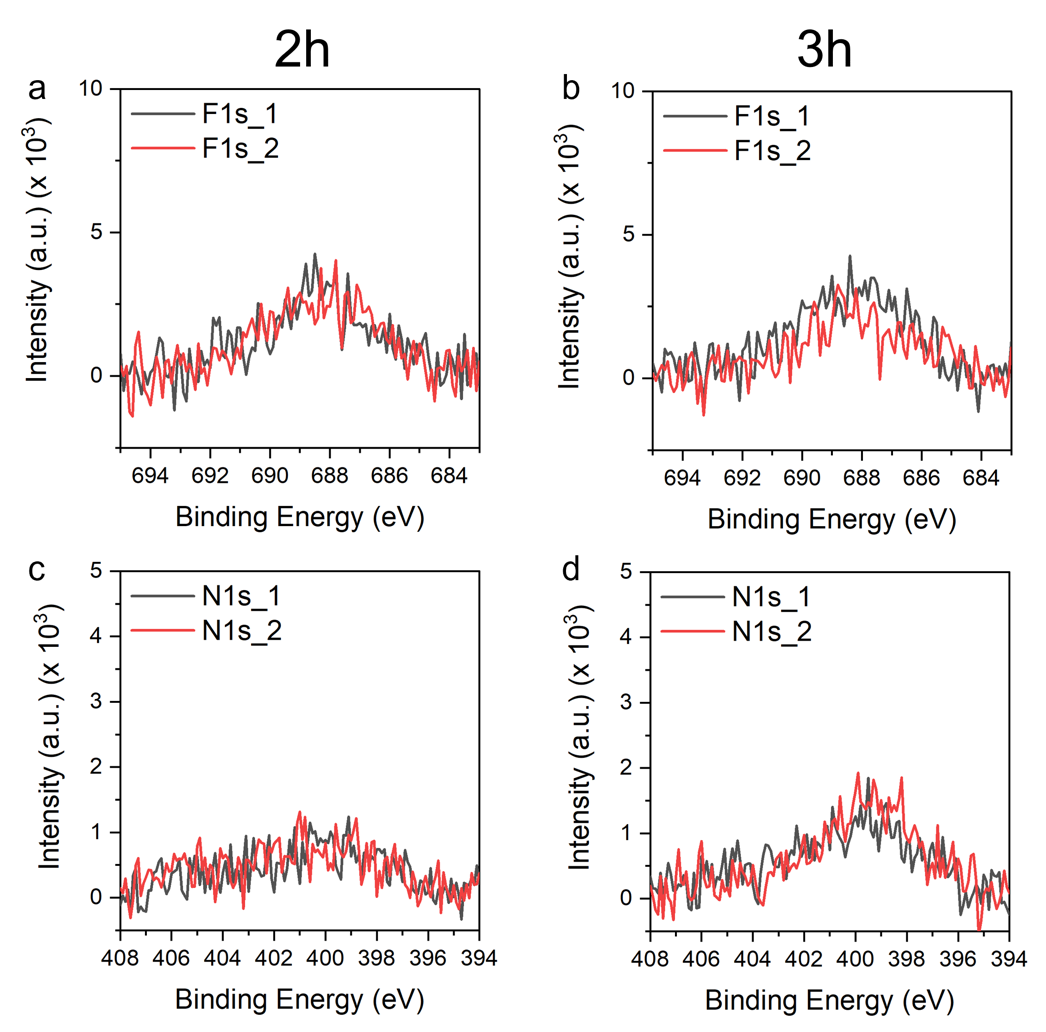
*

*Figure S7. XPS spectra of functionalized graphene in the reaction solution with 3 mM of 4-IBTF, 30 mM of 4-iodoaniline, and 0.3 M of LiPF_6_  for 2h, and 3h, at an applied potential of -1.80 V. (a, b) F1s and (c, d) N1s spectra were measured on two randomly chosen regions in each sample.*

**References**

[1] G. Kresse, J. Furthmüller, *Phys. Rev. B* **1996**, *54*, 11169.

[2] J. P. Perdew, K. Burke, M. Ernzerhof, *Phys. Rev. Lett.* **1996**, *77*, 3865.

[3] S. Grimme, J. Antony, S. Ehrlich, H. Krieg, *J. Chem. Phys.* **2010**, *132*, 154104.

[4] A. V. Krukau, O. A. Vydrov, A. F. Izmaylov, G. E. Scuseria, *J. Chem. Phys.* **2006**, *125*, 224106.
